# Opposite Expression Patterns of *Spry3* and *p75NTR* in Cerebellar Vermis Suggest a Male-Specific Mechanism of Autism Pathogenesis

**DOI:** 10.3389/fpsyt.2019.00416

**Published:** 2019-06-18

**Authors:** Zhenfei Ning, John M. Williams, Romika Kumari, Pavel V. Baranov, Tom Moore

**Affiliations:** School of Biochemistry and Cell Biology, University College Cork, Cork, Ireland

**Keywords:** autism, cerebellum, *SPRY3*, *p75NTR*, pseudoautosomal region, *TMLHE*, carnitine

## Abstract

Autism is a genetically complex neurobehavioral disorder with a population prevalence of more than 1%. Cerebellar abnormalities, including Purkinje cell deficits in the vermis, are consistently reported, and rodent models of cerebellar dysfunction exhibit features analogous to human autism. We previously analyzed the regulation and expression of the pseudoautosomal region 2 gene *SPRY3*, which is adjacent to X chromosome-linked *TMLHE*, a known autism susceptibility gene. *SPRY3* is a regulator of branching morphogenesis and is strongly expressed in Purkinje cells. We previously showed that mouse *Spry3* is not expressed in cerebellar vermis lobules VI–VII and X, regions which exhibit significant Purkinje cell loss or abnormalities in autism. However, these lobules have relatively high expression of *p75NTR*, which encodes a neurotrophin receptor implicated in autism. We propose a mechanism whereby inappropriate *SPRY3* expression in these lobules could interact with TrkB and p75NTR signaling pathways resulting in Purkinje cell pathology. We report preliminary characterization of X and Y chromosome-linked regulatory sequences upstream of *SPRY3*, which are polymorphic in the general population. We suggest that an OREG-annotated region on chromosome Yq12 ∼60 kb from *SPRY3* acts as a silencer of Y-linked *SPRY3* expression. Deletion of a β-satellite repeat, or alterations in chromatin structure in this region due to *trans*-acting factors, could affect the proposed silencing function, leading to reactivation and inappropriate expression of Y-linked *SPRY3*. This proposed male-specific mechanism could contribute to the male bias in autism prevalence.

## Introduction

Autism is a spectrum disorder whose core features include language delay, social deficits, and restricted interests and repetitive behaviours. In addition, there are significant co-morbidities including attention deficit hyperactivity disorder (ADHD), anxiety, intellectual disability, motor delay, and epilepsy, among others (1–3). Recently, the ESSENCE protocol was developed in response to the increasing diagnosis of autism associated with an expanding list of co-morbidities, including cases in which the co-morbidity may be the predominant clinical entity (1, 4). There is also a trend towards the increased diagnosis and inclusion of cases at the mild end of the spectrum (5). The combined effects of these trends may explain the current estimates of autism prevalence in school age children, exemplified by two recent reports, which found 1.68% prevalence in the USA and 2.85% in Northern Ireland (6, 7).

Heritability estimates for autism are high, ranging from 38% (8) to more than 80% (9, 10), and there is an emerging consensus that the majority of the genetic risk is attributable to common genetic variants of small effect size acting in combination with rare or *de novo* variants of larger effect size (11–15).

A striking and unexplained feature of autism is the preponderance of affected males, with a sex ratio of between 3 and 4 to 1 consistently reported, including in recent large studies (6, 7, 16, 17). Earlier studies often reported more extreme male biases, particularly in milder cases (so-called high functioning autism or Asperger’s syndrome), and there is continuing debate on the possibility of a female protective or “camouflage” effect that may result in their under-diagnosis (18–25). Currently, multiple genes and genomic variants are associated with autism with varying levels of confidence; however, the majority are autosomal and do not explain observed sex differences in prevalence (26). Rather than exhibiting sex-specific expression, autism genes may interact with normal regulatory pathways that are themselves sex-specifically regulated (27–29). This is conceptually similar to the proposal that autism genes operate against a background of sex-specific hormone profiles (30–32), and shifts the explanatory burden from the autism genes themselves to the normal sex-specific pathways with which they interact.

We previously analyzed the pseudoautosomal region 2 (PAR2)-linked *SPRY3* gene in autism because it is highly expressed in the cerebellum (33), a region consistently implicated in autism pathogenesis (34–39). *SPRY3* is expressed in Purkinje cells, a key cell type deficient in autism (40, 41), but we note that mouse *Spry3* is not expressed in the cerebellar lobules (VI–VII, X) homologous to those most affected in human autism (33, 41). If this expression pattern is recapitulated in the human, as suggested by a human *SPRY3* promoter–LacZ transgenic mouse strain (33), it suggests two alternative mechanisms by which *SPRY3* could be implicated in loss of Purkinje cells preferentially in these lobules. First, the normal absence of *SPRY3* expression in these lobules may increase their sensitivity to genetic or environmental “insults” that cause Purkinje cell loss. However, this would not explain the male bias. Second, the deregulation and inappropriate overexpression of *SPRY3* in these lobules may be pathogenic, and could provide a male-specific mechanism, as described below. SPRY3 is a receptor tyrosine kinase (RTK) signaling inhibitor that interacts with the TrkB neurotrophin receptor pathway (42), which is implicated in autism and social behavior (43–49).

The X-linked copy of *SPRY3* is adjacent to a known autism gene, *TMLHE*, and a proportion of *SPRY3* transcripts arise from upstream promoters in the X-linked *F8A3* and *TMLHE* regions (33). The *F8A2*–*F8A3* region contains an inversion polymorphism that could potentially affect the expression of flanking genes, including *SPRY3*. The Y-linked copy of *SPRY3* is epigenetically silenced in normal males (50), which could contribute to the male bias in autism due to X-linkage of the expressed gene copy. Alternatively, deregulation and reactivation of the silenced Y-linked copy could provide a male-specific pathological mechanism. A possible further mode of *SPRY3* deregulation is suggested by the fact that *SPRY3* is upregulated in the liver of piglets fed high levels of carnitine (51). Notably, the gene adjacent to *SPRY3*, *TMLHE*, encodes an enzyme in the carnitine biosynthesis pathway. As carnitine deficiency is implicated in autism causation (52), this suggests a mechanism whereby carnitine levels could impact on *SPRY3* regulation and autism.

In this study, we examined the expression of *SPRY3* and its functionally associated genes in cerebellum, and we analyzed genetic variation in predicted X and Y chromosome regulatory regions that may impact on *SPRY3* expression. We propose a pathogenic mechanism in autism involving *SPRY3* deregulation impacting on the BDNF–TrkB–p75NTR neurotrophin pathway.

## Materials and Methods

### Online Bioinformatics and Other Resources

The following databases and online resources were used in this study: UCSC genome browser (https://genome.ucsc.edu/); GENSAT Brain Atlas of gene expression in EGFP Transgenic Mice (http://gensat.org/index.html); Allen Brain Atlases (http://portal.brain-map.org/; 53); GTEx Portal, v7, updated 09/05/2017 (https://gtexportal.org/home/); SFARI (Simon Foundation Autism Research Initiative; https://www.sfari.org/); AGRE (Autism genetic Resource Exchange; https://research.agre.org/program/descr.cfm). Other websites are listed under “Analysis of PsychENCODE data.”

### Whole Mount Immunohistochemistry of Mouse Cerebellum

All reagents were from Sigma, UK, unless otherwise stated. Adult male and female C57Bl/6J mice were humanely euthanized under permissions obtained following animal ethics and welfare review by UCC committees, under national and European legislation. Dissected mouse cerebellum was fixed in 4% Paraformaldehyde (PFA)-Phosphate-buffered saline (PBS) for 10 h, post-fixed in methanol–Dimethyl sulfoxide (DMSO) (4:1) overnight at 4ºC, and then bleached in methanol–DMSO–30% H_2_O_2_ (4:1:1) overnight at 4ºC. After 3 × 60 min wash in 100% methanol, it was frozen at −80ºC and thawed at room temperature (RT) for six cycles in 100% methanol. After rehydrating with 50% methanol, 15% methanol, and PBS for 2 h each, it was digested by proteinase K (10 mg/ml; Sigma, UK) in PBS for 3 min at RT, washed in PBS for 3 × 2 h at RT, and incubated in PBS with 10% goat serum and 0.1% Triton X-100 overnight at 4ºC. It was then incubated with anti-Spry3 primary antibody (Abcam, UK) in PBS containing 10% goat serum, 0.1% Triton X-100, 5% DMSO for 48 h at 4ºC and washed twice in PBS containing 10% goat serum, 0.1% Triton X-100 for 20 min each, followed by incubation with secondary antibody in PBS containing 10% goat serum, 0.1% Triton X-100, 5% DMSO for 24 h at 4ºC. It was then washed twice in PBS containing 10% goat serum, 0.1% Triton X-100 for 2 h each. Immunoreactivity was visualized by incubating the cerebellum in freshly prepared DAB solution (Sigma, UK) for 3 min at RT. The stained cerebellum was imaged with a Nikon SMZ1500 microscope and Nikon DXM1200 camera.

### Droplet Polymerase Chain Reaction Analysis of *F8A2*–*F8A3* Inversion Genotype

All reagents were from Sigma, UK, unless otherwise stated. Cultured cells were lysed in lysis buffer (0.1 M Tris, 0.2 M NaCl, 5 mM EDTA, 0.4% SDS, and 0.2 mg/ml proteinase K, pH 8.0) at 55ºC. Cell DNA was precipitated by adding isopropanol and washed with 70% ethanol. DNA pellet was dissolved in water and digested with *Nru*I and *BspE*I (NEB, UK). Polymerase chain reactions (PCRs) were prepared in a total volume of 100 μl with 1× Go-taq buffer (Promega, UK), 25 mM MgCl_2_, 250 μM dNTPs, 1 μM primers (Eurofins Genomics, Germany): F8A2-F1-2 5′-CACATGATGAAAGTGGGAGGA-3′, F8A2-R2-2 5′-GAATGCAACAAATCAGCAAGA-3′, and F8A2-R3-2 5′-TTCAGACCCATATAGTATTACTGGTGA-3′, 30 nM primer F8A2-R1-2 5′-GCATACACTGCTAGGTGGGAATTCACAGCCACTGGAATGAC-3′, 200 ng digested genomic DNA, and 16 units Go-Taq DNA polymerase.

Emulsion step was carried out by adding PCR reaction dropwise over 30 s to 200 μl light mineral oil with 4.5% v/v Span 80, 0.4% v/v Tween 80, and 0.05% Triton X-100, in a 2 ml Corning Cryo-Tube stirring with a magnetic bar (8 × 3 mm with a pivot ring; VWR) at 1,000 rpm. Emulsions were stirred for 3 min before being overlaid with 30 μl mineral oil. The PCR conditions were 95°C for 120 s; 40 cycles of 95°C for 20 s, 60°C for 30 s, and 72°C for 15 s; 72°C for 5 min. Emulsions were disrupted using 600 μl hexane. Each clean PCR product (2 μl) was amplified in a total volume of 50 μl using primers F8A2-F1-2, F8A2-R2-2, and F8A2-R3-2 and reaction mix: 1× Go-taq buffer, 5 μl 25 mM MgCl_2_, 250 μM dNTPs, 300 nM primers, and 1 unit Go-Taq DNA polymerase.

### Lymphoblastoid Cell Lines and DNA Samples

Cell lines and DNA samples were randomly selected from AGRE (https://research.agre.org/program/descr.cfm) and SFARI (https://www.sfari.org/) resources. AGRE samples are from multiplex families, and SFARI samples are from simplex families. Further details are available from provider websites using sample reference numbers listed below. Additional autism DNA samples were obtained from Prof. David Skuse, University College London. Control DNA samples were from the Caucasian DNA panel from the Coriell Institute for Medical Research, USA. Cells were grown in T25 suspension cell flasks with RPMI-1640 medium supplemented with 10% FBS (Sigma, UK) at 37°C, 5% CO_2_.

Cell lines used were as follows (double-underlining indicates samples with F8A2–F8A3 inversion; see Results section):

AGRE: 2095, 2325, 2396, 2479, 2609, 2615, 2659, 2664, 2718, 2815, 2838, 2853, 2880, 2883, 3126, 2742, 2831, 2327, 2628, 2678, 2326, 2791, 2487, 2328.

SFARI: SSC00317, SSC00591, SSC00636, SSC02727, SSC03440, SSC03459, SSC03537, SSC03774, SSC03989, SSC04232, SSC05124, SSC05350, SSC05435, SSC07444, SSC10172, SSC10210, SSC10777, SSC11067, SSC12271.

### Long-Range PCR of β-Satellite Repeat

PCR reactions were prepared in a total volume of 50 μl with 25 μl 2× GoTaq Long PCR Master Mix (Promega, UK), 10 μl 300 nM primers (Eurofins Genomics, Germany) (Y-Chr BSR-Del-3F 5′-CACAGGCTGTAGTGCAGGTGATG-3′ and Y-Chr BSR-Del-4R 5′-CTGTGTTGTTGATCTGTCTAATGTTGACATTA-3′), and 500 ng genomic DNA. The PCR conditions were 95°C for 120 s; 40 cycles of 93°C for 20 s, 60°C for 16 min; final extension of 72°C for 20 min.

### Analysis of PsychENCODE Data

We obtained paired-end RNA-seq libraries of cerebellar vermis from 33 autism and 38 controls from PsychENCODE (54). Individual libraries contained 50–200 million reads. Human transcriptome sequence was obtained from the RefSeq database (55), downloaded from NCBI (Annotation Release 108). Raw reads were aligned to the set of human RefSeq transcript sequences using bowtie2 short read alignment program (56). Default parameters were used for local alignments. Reads mapping to only one location in the transcriptome were selected by removing the alignments with “XS:i” bowtie2 tag, which represents reads having more than one possible mapping to the reference. SAMtools version 1.3.1 (57) was used to obtain the sorted BAM alignment files, which were further used to predict the heterozygosity in *SPRY3* expressed sequences. *SPRY3* had a total mapped read count range of 653–4033. SAMtools mpileup (57) and BCFtools (58) were used to characterize variations in mapped reads at each coordinate in the *SPRY3* locus. The frequency of variants at each position was analyzed to estimate the likelihood of heterozygosity. For heterozygous genotypes, it is expected that the probability of finding a nucleotide matching the reference sequence at the single nucleotide polymorphism (SNP) position is 0.5, while for homozygous genotypes it is either 0 or 1.

## Results

### Cerebellar Lobule Gene Expression Screen Identifies Opposite Expression of *Spry3* and *p75NTR* in Lobules VI–VII and X

**Figure 1:**
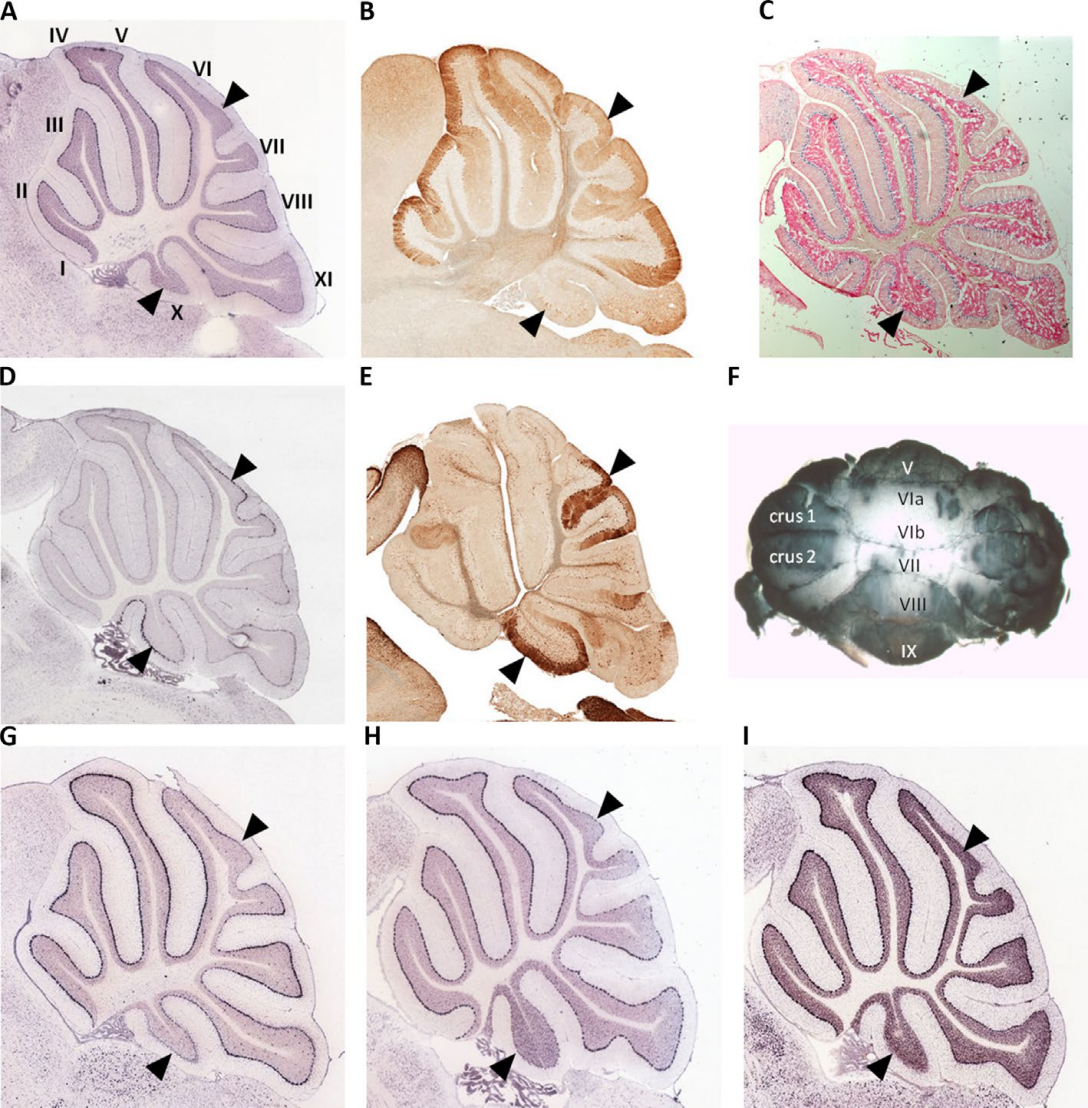
Lobular expression of genes in adult mouse cerebellum. **(A, B)** *Spry3*. **(C)** Human *SPRY3* promoter–LacZ reporter transgenic mouse. **(D, E)** *p75NTR*. **(F)** Representative whole mount immumohistochemistry of adult female mouse cerebellum using anti-Spry3 antibody. **(G–I)** *Abhd3*, *Lrp8*, and *Plcβ4*. Images **(A, D)** and **(G–I)** were from Allen Brain Atlas; **(B** and **E)** were from GENSAT. Data for images **(A–C)** were previously published (31) and are included here for comparison with p75NTR expression. Roman numerals (I–X) in panel A indicate lobule identity. Arrowheads indicate lobules VI–VII and X with notable gene expression patterns.

We used whole mount immunohistochemistry of cerebellums from adult male and female C57Bl/6J strain mice and confirmed relatively low Spry3 expression in lobules VI–VII and X, as previously noted in mouse Allen Brain Atlas (ABA) and GENSAT data [**Figure 1A, B, F**; see also Ref. (33)]. We next sought to determine whether other genes share this expression pattern by visually inspecting the spatial expression patterns of genes in sagittal sections of the mouse ABA data as follows: i) 54 genes with biased expression in “Cerebellar cortex, Purkinje layer” under the “Fine Structure Search” option of the mouse ABA (**Supplementary Table 1**); ii) mouse homologues of 87 high-risk autism genes from SFARI (https://www.sfari.org/resource/sfari-gene/#bottom; **Supplementary Table 2**). Three of 54 cerebellar cortex-biased gene set (*Abhd3*, *Lrp8*, and *Plcβ4*) had lower expression in lobules VI–VII and X (**Figure 1G, H, I**), reminiscent of the *Spry3* pattern, but none of 87 SFARI gene mouse homologues had this pattern; however, many of the latter had faint staining and were difficult to score.

**Figure 2:**
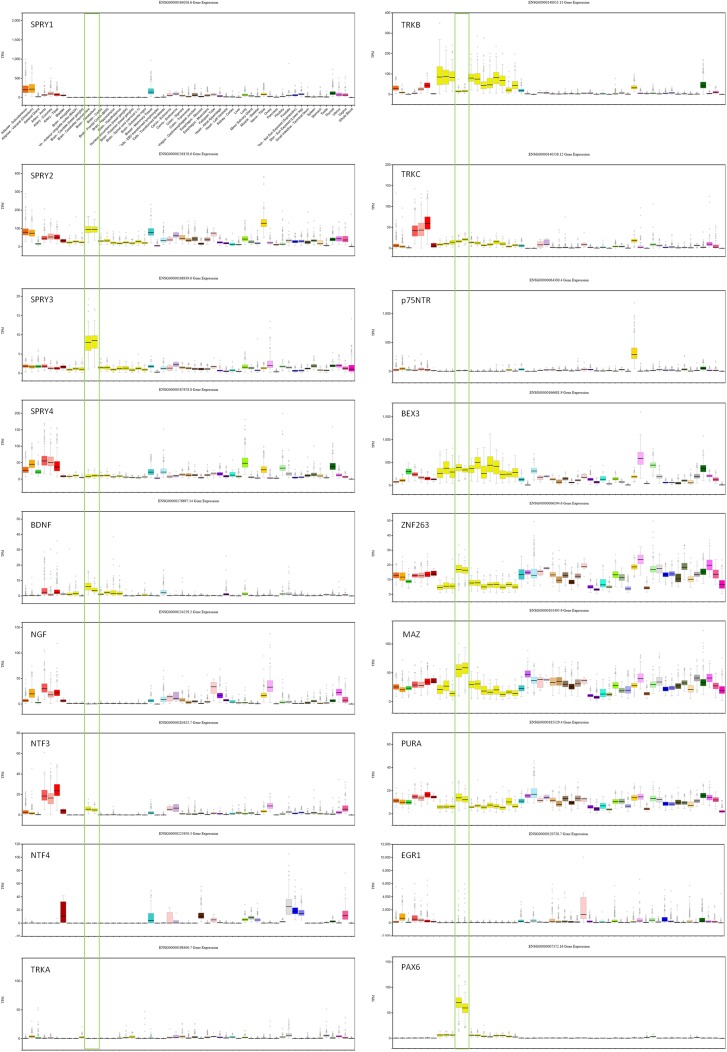
GTEx data for genes associated with *SPRY3* expression and regulation. Cerebellum samples are boxed to highlight expression relative to other tissues: brain–cerebellar hemisphere; brain–cerebellum. Note different scales on TPM (transcripts per million) Y-axis for each gene.

Transcription factors (TFs) predicted to regulate human *SPRY3* (ZNF263, MAZ, PURA, EGR1, PAX6) are expressed in mouse Purkinje cells (33), and GTEx data confirm relatively high expression of these factors in human adult cerebellum (**Figure 2**). However, limited data on these genes in ABA and GENSAT did not allow us to determine whether their spatial expression patterns coincide with *Spry3* lobular expression.

We next examined spatial expression of *Spry1*, *Spry2*, and *Spry4*; neurotrophins (*Ngf*, *Bdnf*, *NTF3*, and *NTF4*); neurotrophin receptors (*p75NTR*, *TrkA*, *TrkB*, and *TrkC*); and *Bex3* (*Ngfrap1*), which encodes a p75NTR interacting protein (59), in cerebellum to determine possible lobular co-expression with *Spry3*. On ABA, *Spry1* and *Spry4* exhibit faint staining, whereas *Spry2* is widely expressed, including in cerebellar Purkinje cells, but does not exhibit a specific lobular expression pattern like *Spry3*. This is consistent with GTEx data in which *SPRY2* has relatively high expression in human cerebellum, whereas *SPRY1* and *SPRY4* exhibit no and low expression, respectively (**Figure 2**).

For the neurotrophins, expression data on GTEx indicated that *BDNF* and *NTF3* are relatively highly expressed in cerebellum, compared to other brain regions, whereas there was no expression of *NGF* and *NTF4* (**Figure 2**). However, on ABA, mouse *Ntf4* is expressed in Purkinje cells, whereas there is no *Bdnf *or* Ngf*, and barely detectable *Ntf3* expression. It is unclear if these species differences reflect biological differences or technical limitations.

The data for neurotrophin receptor expression were generally consistent between ABA (mouse) and GTEx (human) datasets. On ABA, *TrkB* is widely expressed in the brain, including in cerebellar Purkinje cells, and GTEx data also indicated its wide expression in brain including cerebellum (**Figure 2**). *TRKA* and *TRKC* exhibited low and moderate expression, respectively, on GTEx, and no expression was detected on ABA. Analysis of *p75NTR* expression in GTEx suggested that it is virtually absent from the central nervous system, apart from a marginal signal in cerebellum and high expression in the peripheral nervous system (spinal cord, tibial nerve; **Figure 2**). However, scrutiny of ABA and GENSAT data indicated wide expression of *p75NTR* in the mouse adult brain, but with restricted expression in cerebellar vermis Purkinje cells, largely restricted to lobules VI–VII and X (**Figure 1D, E**), the exact opposite of the *Spry3* lobular expression pattern. *Bex3* (and other *Bex* genes; data not shown) is expressed throughout the adult mouse brain (ABA), including in cerebellar Purkinje cells, and is highly expressed in human cerebellum (**Figure 2**).

### Genetic Analysis of X Chromosome-Linked Regulatory Sequences Upstream of Human *SPRY3*

**Figure 3:**
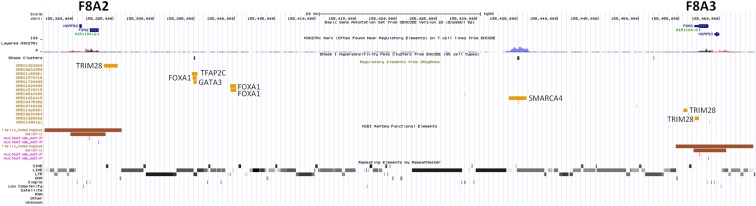
ENCODE/OREG annotations in Xq28 F8A2–F8A3 interval.

**Figure 4:**
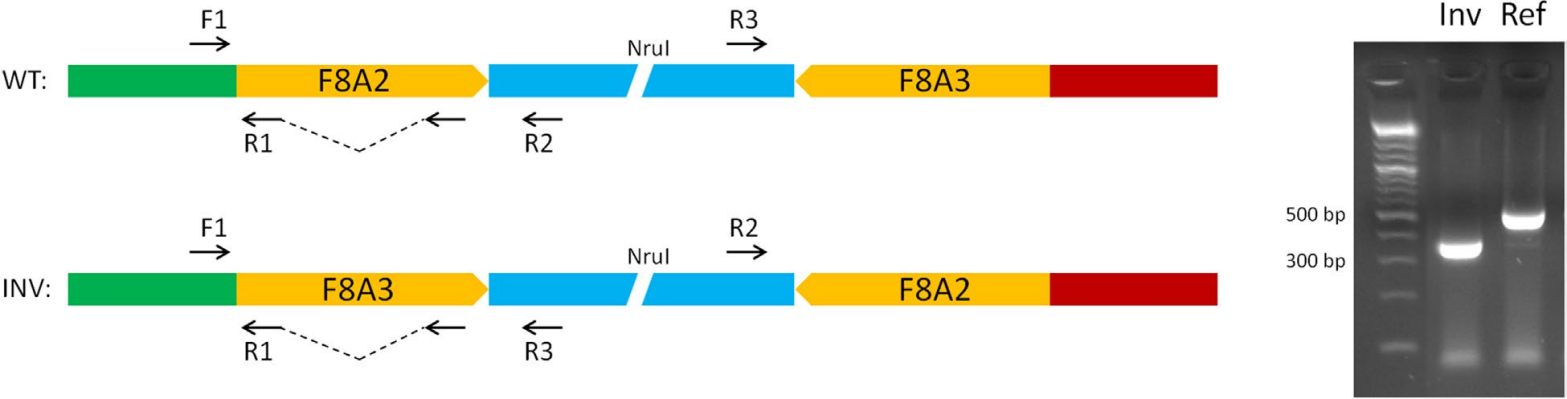
Strategy for single-molecule PCR genotyping of F8A2–F8A3 inversion polymorphism. Ref, orientation allele described in reference sequence on UCSC browser (HumanGRch37/hg19); Inv, opposite orientation to reference sequence.

The unique genomic configuration of human *SPRY3* due to the evolution of the PAR2 in the hominin lineage suggests that sex-linked upstream elements could regulate expression of X-linked *SPRY3* and epigenetic silencing of Y-linked *SPRY3* (33). X-linked *SPRY3* transcription initiates in the *F8A3*–*TMLHE* region (33). *F8A3* is associated with inversions involving *F8A1* and *F8A2* (60, 61). The *F8A2*–*F8A3* interval contains regulatory elements approximately 20 and 60 kb centromeric of F8A3 (**Figure 3**). Inversion of this sequence might affect regulation of flanking genes including *F8A3* region-associated *SPRY3* transcription. We developed a single-molecule droplet PCR assay to determine the orientation of the *F8A2*–*F8A3* interval using a modified version of Turner et al. (62, 63) (**Figure 4**). We analyzed DNA from autism cases comprising 20 individuals from SFARI resource and 24 individuals from AGRE, representing simplex and multiplex families, respectively (see Materials and Methods for sample identifiers). There were 2/20 and 4/24 inversions compared to the reference sequence (GRCh38/hg38 assembly, December 2013, UCSC browser), which is similar to the 20% frequency (4/20 samples) found in non-autistic *F8* gene-associated hemophilia patients (64).

### Analysis of Y Chromosome-Linked Regulatory Sequences Upstream of Human *SPRY3*

**Figure 5:**
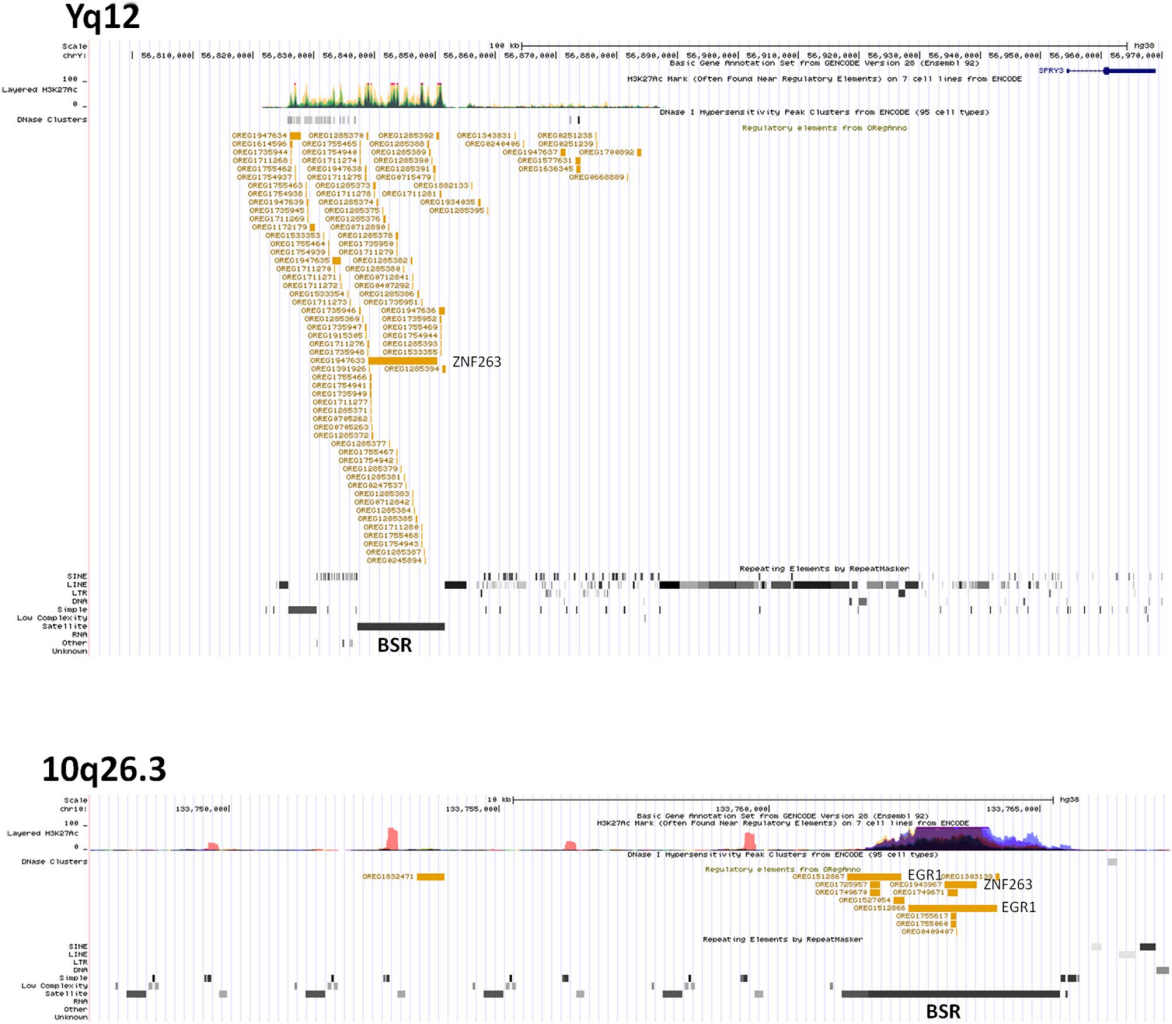
ENCODE/OREG annotations in Yq12 and 10q26.3 regions showing major DNA factors binding in BSR regions (ZNF263, EGR1). Note different scales in each panel.

We hypothesised that Y chromosome sequences proximal to the Yq–PAR2 boundary act as a silencer of Y-linked *SPRY3*. Scrutiny of this region using the UCSC browser identified a predicted regulatory region that begins 60 kb upstream of the *SPRY3* PAR2 transcriptional start site (TSS) and extends a further 60 kb towards the centromere. This element comprises compositionally distinct regions of ∼25 and ∼35 kb and is flanked by a 50 kb sequence gap proximally, on the far side of which are the major Yq12 satellite sequences (**Figure 5**). The 25 kb region comprises ∼10 kb of simple CATTC and CACTC repeats, while the remaining ∼15 kb is a beta satellite repeat (BSR). The entire ∼60 kb region has multiple *DNase*I sensitive sites and CTCF binding sites (data not shown), and the BSR, similarly to the *SPRY3* core promoter AG-rich repeat, contains multiple ZNF263 binding sites. BLAST of the ∼15 kb Yq12 BSR identified related sequences on chrs. 4, 10, 14, and 18 with 100% coverage and identity scores of 78–83%. The BSR-containing allele of the *FSHD* gene locus on chr. 4q35 (65) is not represented on the UCSC browser; but the structurally similar 10q26.3 locus is (66, 67). This exhibits a different pattern of chromatin modifications to the Yq12 BSR, increasing our confidence in the attribution of Yq12 annotations (**Figure 5**).

**Figure 6:**
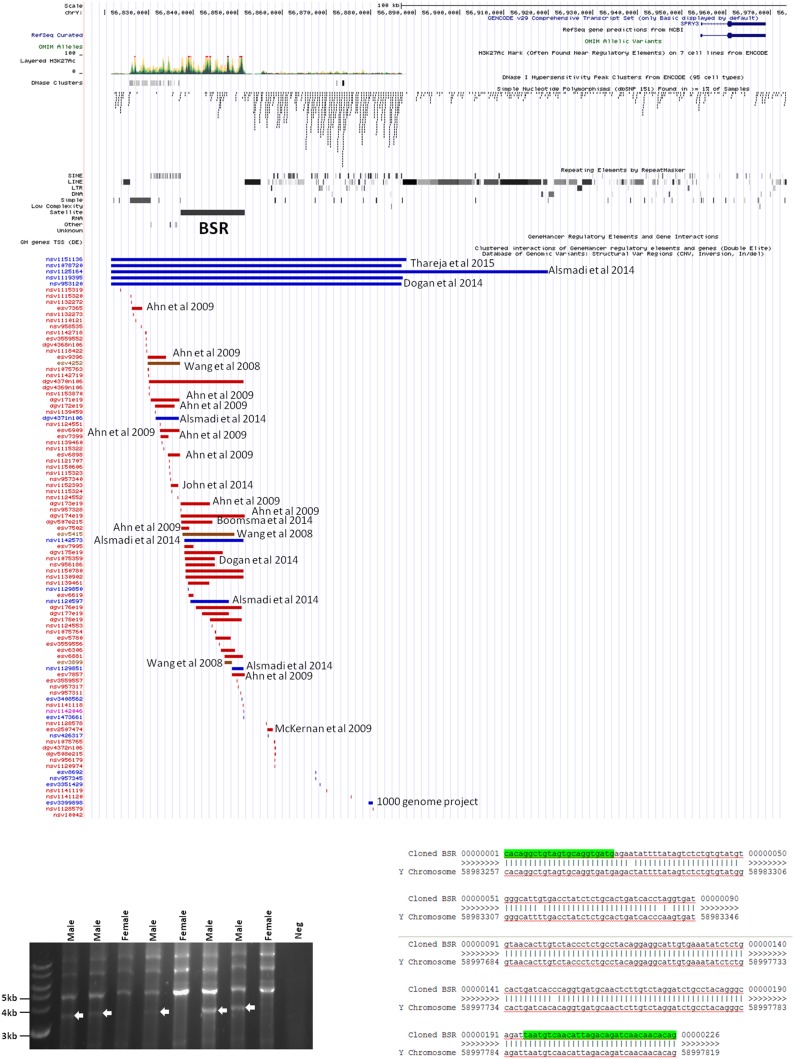
**(A)** Map of genomic variants in Yq12 region. Details of annotated publication references are available on UCSC browser. **(B)** Male-specific ∼3.5 kb PCR product amplified by BSR-specific primers (8 of 57 samples analyzed are shown). **(C)** Sequences of 5′ and 3′ ends of cloned PCR product.

Inspection of the Yq12 region in the database of structural variants (DSV) using the UCSC browser indicated significant variability of the BSR, including independently reported deletions and duplications (**Figure 6A**). We attempted to use long PCR of genomic DNA using primers flanking the BSR to determine whether structural variants or length polymorphisms are associated with autism. Primer design was severely restricted due to the genomic architecture of the region, and the selected primer pair amplified a ∼3.8 kb product from all male samples and no female samples (**Figure 6B**). Samples were as follows: 20 male autism (AGRE); 21 male autism (Skuse samples); 12 normal male and 4 normal female (Skuse and Coriell Caucasian panel). Cloning of this PCR product was problematic, but sequences obtained from both ends of a cloned partial product matched the expected 5′ and 3′ boundaries of the reference sequence (**Figure 6C**). However, this product was not amplified from BAC clone RP11-88F4, which covers the region, reducing confidence in the genomic location of the template for the ∼3.8 kb amplicon (data not shown).

### Analysis of *SPRY3* Allele-Specific Expression in Cerebellum Using PsychENCODE Dataset

**Figure 7:**
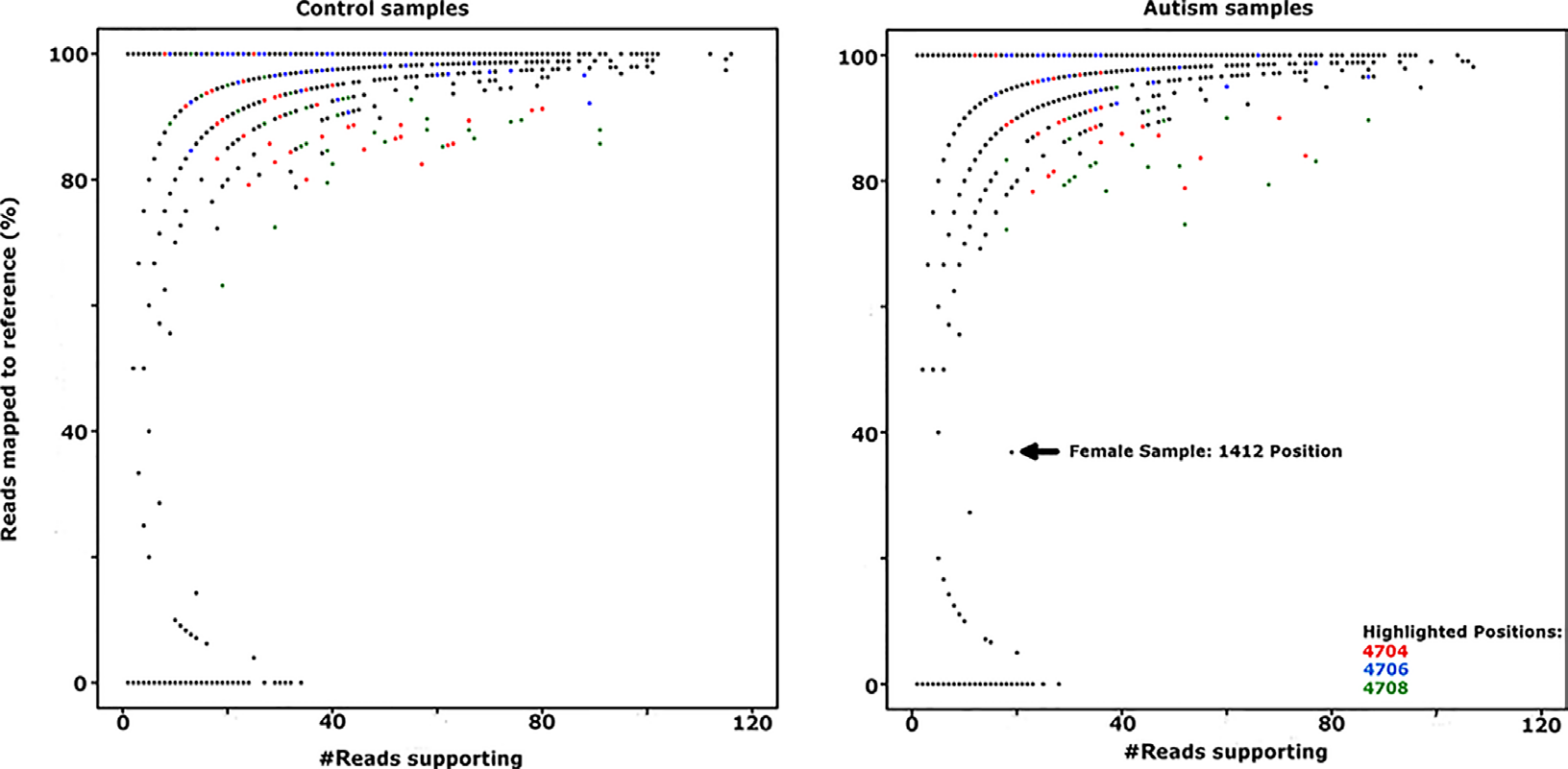
Comparison of *SPRY3* (RefSeq ID: NM_005840) expression heterozygosity maps for autism and control samples. SNP positions 4704, 4706, and 4708 (highlighted in red, blue, and green, respectively) produced all possible genotypes in the majority of control and autism samples, suggesting a sequencing artefact at these positions, and were not counted as heterozygotes. Female sample 1412 position is included as indicative of a true heterozygous sample.

Genetic and structural analysis of the Yq12 putative regulatory region was inconclusive; therefore, we looked for loss of epigenetic silencing and reactivation of the Y-linked *SPRY3* allele in a comparison of RNA-Seq data from male autism and control cerebellum samples. We obtained paired-end RNA-seq libraries of cerebellar vermis from 33 autism and 38 control samples from PsychENCODE (54). Genotypes for X and Y chromosome (including PAR2) markers are not available for these samples; therefore, we used a statistical approach to analyze the level of heterozygosity of transcripts of the *SPRY3* and control genes. Heterozygous expression of *SPRY3* would be indicative of expression of both X- and Y-linked alleles due to pathological reactivation of the Y-linked copy. Genes flanking *SPRY3* were also examined. *TMLHE *was used as a negative control to estimate the level of variants due to technical noise (e.g., sequencing errors, substitutions during library preparation, and misalignments) since it is X-linked and no bona fide heterozygosity is expected. *SYBL1* was analyzed because it flanks *SPRY3* distally and is similar to *SPRY3* in having a Y-linked copy; however, its silencing is associated with a methylated CpG island, and it may be regulated differently to *SPRY3* (68). Autosomal genes (*NPTN* and* MCM6*) were used as positive controls, where we expected to identify heterozygosity. There was no significant difference in the heterozygosity plots of autism versus control samples for any of the genes analyzed (**Figure 7**; **Supplementary Figure 1**).

## Discussion

We have extended our previous work implicating *SPRY3* in autism (33) and provide evidence for a possible mechanism of chromosome Y-linked *SPRY3* gene deregulation underpinning male susceptibility. At the cellular level, we propose that *SPRY3* deregulation affects the functioning of the BDNF–TrkB–p75NTR neurotrophin pathway, leading to cerebellar Purkinje cell pathology. Our hypothesis can explain the specific lobular distribution of Purkinje cell loss in autism (lobules VI, VII, and X), as previously described (41). More speculatively, *SPRY3* deregulation could explain a reported, although currently unconfirmed, lung branching abnormality in autism (69).

The male bias in autism prevalence is not explained by known DNA susceptibility variants because the majority are autosomal, and a major sex-linked gene effect has not been identified (10, 14, 17, 26, 70–76). This suggests that autosomal variants interact with one or more sex-specific developmental or regulatory pathways, which could include sex hormones, or X or Y chromosome-linked gene-encoded regulators. This hypothesis requires that the majority (perhaps hundreds) of individual susceptibility variants converge on sex-specific mechanisms that underpin either a female protective effect or male susceptibility effect (FPE or MSE) (27, 29). The plausibility of FPE/MSE mechanisms is supported by mouse mutants of known autism genes, which exhibit sex-specific phenotypes (77–79), the presence of X-linked regulators expressed differently in males and females (80), and the influence of sex hormones such as testosterone and estrogen on normal and abnormal brain development and function (30, 31, 81–83).

However, recent studies did not detect a predicted Carter effect in autism because an increase in disease aggregation in families with a female proband was not observed, as would be expected if affected females require a higher mutation burden to overcome an FPE threshold (73, 84–86). This suggests that sex-specific departures from normal physiology, rather than normal sex-specific physiology *per se*, may underlie the male bias in autism (87). An alternative hypothesis to FPE/MSE is therefore the existence of one or more male-specific disease mechanisms (MDM). Such MDM would have to occur at a high frequency to explain the large male prevalence bias.

There are potentially two general mechanisms of *SPRY3* deregulation in autism. First, *trans*-acting effects of susceptibility variants at other loci encoding, for example, chromatin regulators could deregulate X- or Y-linked *SPRY3*. This category would also include environmental effects, for example, due to alterations in carnitine levels, to which *SPRY3* may be responsive (see below). Second, *cis*-acting *de novo* mutations or common variants in regulatory regions could cause aberrant expression of X- or Y-linked *SPRY3*. Previous genetic studies have not associated the Xq28 or PAR2 regions with autism (88). However, both the X and Y chromosome regions upstream of PAR2 are structurally complex and difficult to analyze, and therefore, autism-associated variants may have been overlooked.

The *F8A2*–*F8A3* interval has a common inversion polymorphism that may alter the orientation and distance from *SPRY3* of ENCODE/OREG-predicted regulatory sequences. The major regulatory factors that bind in this region (TRIM28, SMARCA4) are associated with autism (89–91); therefore, inversions or other rearrangements of this region may impact on expression of F8A2/F8A3 region-associated transcription or on flanking genes (*CLIC2*, *TMLHE*, *SPRY3*), potentially contributing to autism risk. It is unknown how frequently *de novo* inversions occur, and inversion alleles are not tagged by known SNPs. Therefore, we used single-molecule analysis to determine orientation of this inversion in a small number of autism and control DNA samples. We observed similar allele frequencies to those reported from an analysis of 20 hemophilia patients (64), with no evidence of a strong association with autism.

The Yq12 PAR2 region is poorly characterized due to the absence of genetic recombination and the highly repetitive DNA sequences that comprise much of the Yq and PAR2 boundary regions. However, our identification of a putative regulatory region in distal Yq12, 60 kb upstream of the *SPRY3* TSS, suggests a mechanism of silencing of Y-linked *SPRY3*. Similar to the *SPRY3* core promoter AG-rich repeat (33), a BSR in this region has multiple ZNF263 binding sites, suggesting a possible regulatory interaction with the *SPRY3* promoter. A BSR at chr. 10q26.3, also annotated in ENCODE, has a different pattern of chromatin modifications, increasing confidence in the Yq12 annotations. Interestingly, copy number variants (CNV) in the 10q26.3 region are associated with autism, although there is no evidence that this is due to the BSR (92; https://gene.sfari.org/database/cnv/10q26.3). Non-BSR sequences in the Yq12 region have abundant CTCF binding sites, a factor associated with gene imprinting and genome topology (93), further suggesting a regulatory function for this region in regulating Y-linked *SPRY3*.

There is extensive sequence and structural variation across the Yq12 putative regulatory region, including reported length polymorphisms of the BSR. However, BSRs are abundant in the genome (94, 95), and the majority are not annotated; therefore, caution is required when interpreting annotations arising from genome-wide studies underpinned by short sequence reads. We were unable to confirm Yq12 BSR variation using long PCR due to severe sequence constraints in primer design and instability of cloned PCR products. Other approaches, such as fiber FISH or single-molecule sequencing, anchored in unique sequences in PAR2, will be required to provide confirmation of BSR length alleles and to conduct genetic association studies. We also cannot exclude a role for somatic cell mutations, which are increasingly implicated in neurodegeneration (96). Somatic instability of the repeat-rich Yq12–PAR2 region could result in cell autonomous DNA rearrangements and deregulation of Y-linked *SPRY3*.

Notwithstanding significant technical difficulties in analyzing the Yq12–PAR2 boundary region, our data suggest a hypothetical mechanism whereby genetic (DNA sequence) or epigenetic (chromatin structure) variation could lead to reactivation and inappropriate lobular expression of Y-linked *SPRY3*. We sought to test this hypothesis by analyzing allelic expression of *SPRY3* in PsychENCODE cerebellum expression data, but we did not detect biallelic expression in male samples as would be predicted if the Y-linked copy is active. However, we lacked the sample genotypes and information about the exact cerebellar lobules sampled. Also, the pathological mechanism we propose may not be detectable in RNA-Seq data if reactivation of Y-linked *SPRY3* ultimately results in Purkinje cell death. Therefore, we do not consider this a definitive rejection of our hypothesis.

A possible further mode of *SPRY3* deregulation is suggested by its linkage with *TMLHE* and their overlapping regulatory sequences (33). TMLHE is an enzyme in the carnitine biosynthesis pathway, and carnitine deficiency is associated with autism (52, 97, 98). Intriguingly, there is evidence that carnitine levels modulate *SPRY3* expression in the pig (51). *TMLHE* mutations are rare but well-established autism susceptibility factors (52, 88, 97, 99, 100). Mouse *Tmlhe* is expressed in Purkinje cells; however, the lobular expression pattern is not restricted like *Spry3*. At least one mutation attributed to *TMLHE*-associated autism risk also affects *SPRY3* sequences (33), suggesting a possible role for deregulation of *SPRY3* in some reported cases.

Deficits in cerebellar vermis structure and Purkinje cell number and morphology have been reported frequently in autism at post mortem, using MRI imaging, and in mouse models (39, 82, 101–110). In a histological study of human brain tissues from autism cases, Skefos et al. (41) reported that Purkinje cell loss predominantly affects crus I and II (lobule VIIa), and they also noted a possible male-specific deficit in lobule X of the flocculonodular lobe. Following our previous study (33), and arising from our current observations, we show that mouse *Spry3* and *p75NTR *have opposite expression patterns in cerebellar vermis lobules VI–VII and X. *Spry3* is not expressed in these lobules, whereas *p75NTR* is strongly expressed [see also **Figure 2** in Ref. (111) and **Figure 1F** in Ref. (112)]. In a screen of 135 genes in the adult mouse (selected for high cerebellar expression or prior association with autism), we identified only three (*Abhd3*, *Lrp8*, and *Plcβ4*) with a somewhat similar lobular expression pattern to *Spry3*, and none that recapitulated the *p75NTR* expression pattern. This suggests that there are relatively few genes whose expression could explain the lobular pattern of abnormalities described by Skefos et al. (41). Our qualitative screen of the ABA mouse data was restricted to adult brain and may therefore lack sensitivity; however, we are reassured regarding its specificity by an independent report that *Plcβ4* is not expressed in lobules VI–VII and IX–X (113).

The opposite expression patterns of *Spry3* and *p75NTR* mirror the well-known opposite expression patterns of *zebrin II*/*aldolase C* and *Hsp25*/*Hspb1* on the anterior–posterior (AP) axis (114–118). The mechanisms responsible for patterning the anterior–posterior axis of the cerebellum include the autism gene *Engrailed-2* (*En2*; 118, 119), and intriguingly, *En2* has specific functions in the development of lobules VI–VII and X (118, 120), suggesting a further mechanism underpinning involvement of these lobules in autism (after 39).

A recent report suggests that lobules VI–VII (Crus I) in rodents are homologous to Crus I and II in primates (121). Therefore, if mouse *Spry3* and *p75NTR* expression patterns are conserved in human, as appears likely from a *SPRY3* promoter–reporter transgenic mouse (33), and scrutiny of the ABA (human) and GTEx databases, we can propose an MDM in which aberrant expression of human *SPRY3* in lobules VI–VII and X interferes with neurotrophin signaling, causing Purkinje cell pathology through a BDNF–TrkB–p75NTR mechanism. Spry1, 2, and 4 regulate receptor tyrosine kinase signaling including FGFR (122–125), whereas evidence from *Xenopus* and mouse indicates a regulatory loop involving BDNF- and TrkB-dependent expression of *Spry3*, and Spry3-mediated inhibition of BDNF–TrkB signaling (42). Therefore, it is possible that both SPRY3 and p75NTR proteins interact with BDNF–TrkB signaling, a pathway implicated in neuronal (including Purkinje) cell development and survival (126–130). Although *p75NTR* is not listed on the SFARI autism gene database, it is a compelling candidate for involvement in autism pathogenesis (128, 131, 132). There is extensive evidence of both pro- and anti-apoptotic functions for p75NTR, particularly in contexts with concomitant alteration of neurotrophin receptor signaling, including of TrkB (130, 133–138). SPRY3 regulates TrkB signaling (42); therefore, we speculate that inappropriate expression of SPRY3 in lobules VI–VII and X, in the context of TrkB and p75NTR expression, may affect Purkinje cell function or survival, although the exact mechanism would have to be established in relevant models, as neurotrophin signaling effects depend strongly on physiological context (136, 139).

**Figure 8:**
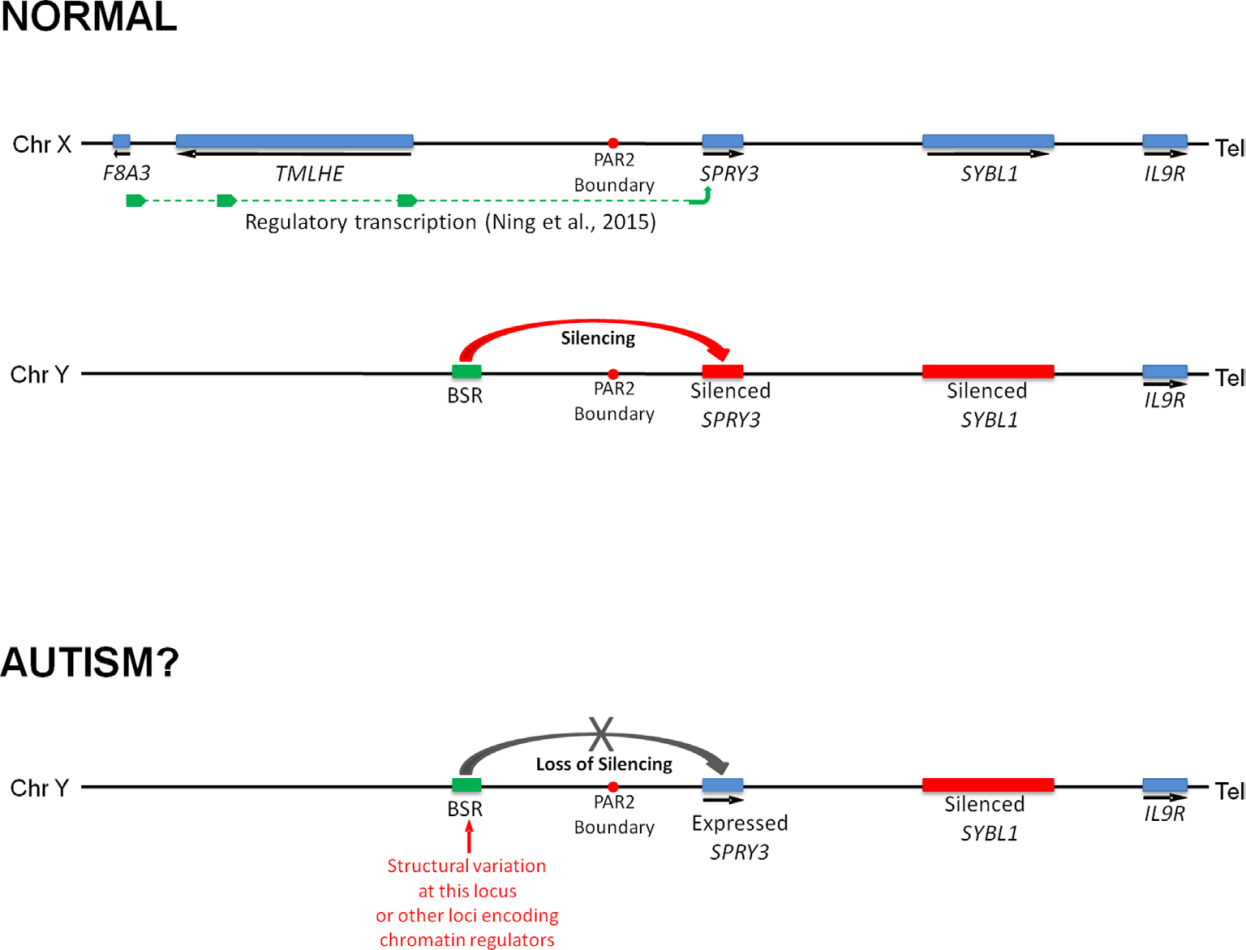
Model of regulation of human *SPRY3* by X and Y chromosome-linked regulatory regions and deregulation in autism. We propose that regions including the *F8A2*–*F8A3* interval and associated transcription spanning the Xq28–PAR2 boundary, and Y-linked ENCODE/OREG-annotated regions including the BSR, may be deregulated by *cis*- or *trans*-acting factors. More specifically, we propose that cis-acting Yq12 genomic variants, including BSR length variants, may result in variable reactivation and expression of Y-linked *SPRY3*, leading to inappropriate lobular expression. Y-linked transcription may have altered lobular expression due to lack of putative Xq28-linked regulatory elements associated with the normal pattern of lobular expression from X-linked *SPRY3* or due to influence of Yq12 genomic elements.

We previously reported that a human *SPRY3* promoter–LacZ reporter transgenic mouse substantially recapitulated the mouse *Spry3* expression pattern (33). Similar to mouse *Spry3*, human *SPRY3*-LacZ is expressed in Purkinje cells throughout the cerebellum, except in lobules VI–VII. However, unlike mouse *Spry3*, human *SPRY3*-LacZ is expressed in lobule X. Therefore, sequences outside of the human core promoter, or *trans*-acting factors whose expression differs between mouse and human, may be required for regulation of *SPRY3* in this lobule. Interestingly, the deficits in lobule X identified by Skefos et al. (41) may be male-specific, which, in the context of our proposed mechanism of pathological over-expression of *SPRY3*, could indicate a role for deregulation (reactivation) of the Y-linked copy in this lobule, which might have a different expression pattern compared to the X-linked copy due to the lack of cis-acting X-linked regulatory sequences, or the inappropriate influence of Y-linked sequences (**Figure 8**).

Finally, we note that Sprouty was originally described based on a branching phenotype of the apical airways of Drosophila (140), and mouse* Spry2* coordinates vascular and airway branching in the lung (141). *Spry3* is expressed in the mouse lung bronchial tree (our unpublished data) and in human lung (GTEx), suggesting that deregulation of *SPRY3* could potentially provide a mechanism underpinning a lung branching abnormality reported in autism patients (69).

In future work, we aim to deepen our understanding of *SPRY3* and *p75NTR* expression and functional interactions during brain and lung development in the human and mouse, including in autism mouse models. Transgenic under- or over-expression of *Spry3* in cerebellar lobules VI–VII and X in mice would provide an *in vivo* model of our proposed MDM in autism. Due to the unique genomic architecture and regulation of the human PAR2, and the difficulty in sourcing matched tissue samples from specific cerebellar lobules from normal and autism brains, and from other organs such as lung, the analysis of Y-linked *SPRY3* deregulation in the cerebellum and lung will be challenging, particularly if the pathology results in cell death. However, advances in single-molecule DNA sequencing techniques will facilitate detection of genomic variants in this region that may be associated with autism.

## Ethics Statement

Access to human genetic data and biomaterials for this study was carried out in accordance with the procedures and recommendations of the UCC Office of the Vice-President for Research and Innovation, specifically for accessing AGRE and SFARI datasets and biomaterials, under their respective procedures. All subjects gave written informed consent in accordance with AGRE and SFARI protocols and procedures, consistent with the Declaration of Helsinki. Access to animal tissues for this study was pursued in accordance with the recommendations of the UCC Animal Experimentation and Ethics Committee under licencing from the Irish Health Products Regulatory Authority (https://www.hpra.ie/).

## Author Contributions

ZN and JW carried out all of the bench work and aspects of bioinformatics and data analysis. RK and PB carried out analysis of PsychENCODE data. TM conceived of, and supervised, the study and wrote most of the manuscript. All authors contributed to various aspects of manuscript preparation.

## Funding

This work was funded by grants to TM from Science Foundation Ireland (TIDA Award 13/TIDA/B2659) and Higher Education Authority “Irish Transgenic Network” award.

## Conflict of Interest Statement

The authors declare that the research was conducted in the absence of any commercial or financial relationships that could be construed as a potential conflict of interest.

## Supplementary Material

Supplementary Table 1 Mouse Purkinje cell expressed genes whose spatial expression was examined in Allen Brain Atlas data. For consistency, the numbering scheme for the 54 genes identified as “cerebellum, Purkinje cell enhanced” in the Allen Brain Atlas “fine structure” search is maintained here, with inclusion of two blank rows (29 and 53).

Supplementary Table 2 Human autism genes from SFARI “High confidence” and “Strong candidate” lists (https://www.sfari.org/resource/sfari-gene/#bottom) for which spatial expression of their mouse orthologues was examined in Allen Brain Atlas data.

Supplementary Figure 1 Comparison of SPRY3 expression heterozygosity map with maps for TMLHE (X-linked), SYBL1 (PAR2-linked), and NPTN, MCM6 (autosomal) genes.
