# Supplementary material for: Opposite Expression Patterns of Spry3 and p75NTR in Cerebellar Vermis Suggest a Male-Specific Mechanism of Autism Pathogenesis
Source: Front Psychiatry. 2019 Jun 18;10:416. doi: 10.3389/fpsyt.2019.00416 (PMC6591651; doi:10.3389/fpsyt.2019.00416)
Supplement: Supplementary Table 1 — Mouse Purkinje cell expressed genes whose spatial expression was examined in Allen Brain Atlas data. For consistency, the numbering scheme for the 54 genes identified as “cerebellum, Purkinje cell enhanced” in the Allen Brain Atlas “fine structure” search is maintained here, with inclusion of two blank rows (29 and 53). [file DataSheet_1.docx]

| **Genes identified as ‘cerebellum, Purkinje cell enhanced’ in Allen Brain Atlas ‘fine structure’ search**  Supplementary Table 1. | |
| --- | --- |
| **Rank** | **Gene** |
| 1 | *Calb1*  *calbindin 1* |
| 2 | *Cacna1g*  *calcium channel, voltage-dependent, T type, alpha 1G subunit* |
| 3 | *Grid2*  *glutamate receptor, ionotropic, delta 2* |
| 4 | *Kcnc3*  *potassium voltage gated channel, Shaw-related subfamily, member 3* |
| 5 | *Id2*  *inhibitor of DNA binding 2* |
| 6 | *Rgs8*  *regulator of G-protein signaling 8* |
| 7 | *Cpne2*  *copine II* |
| 8 | *Ppp1r17*  *protein phosphatase 1, regulatory subunit 17* |
| 9 | *Fam107b*  *family with sequence similarity 107, member B* |
| 10 | *Pcsk6*  *proprotein convertase subtilisin/kexin type 6* |
| 11 | *Lancl1*  *LanC (bacterial lantibiotic synthetase component C)-like 1* |
| 12 | *0610007P14Rik*  *RIKEN cDNA 0610007P14 gene* |
| 13 | *Erp29*  *endoplasmic reticulum protein 29* |
| 14 | *Inpp5a*  *inositol polyphosphate-5-phosphatase A* |
| 15 | *Abhd3*  *abhydrolase domain containing 3* |
| 16 | *Grid2ip*  *glutamate receptor, ionotropic, delta 2 (Grid2) interacting protein 1* |
| 17 | *Hpcal1*  *hippocalcin-like 1* |
| 18 | *Dbi*  *diazepam binding inhibitor* |
| 19 | *Arhgap26*  *Rho GTPase activating protein 26* |
| 20 | *Dpp10*  *dipeptidylpeptidase 10* |
| 21 | *Cst3*  *cystatin C* |
| 22 | *Ryr1*  *ryanodine receptor 1, skeletal muscle* |
| 23 | *Steap2*  *six transmembrane epithelial antigen of prostate 2* |
| 24 | *Gabbr2*  *gamma-aminobutyric acid (GABA) B receptor, 2* |
| 25 | *Cerk*  *ceramide kinase* |
| 26 | *Lrrn2*  *leucine rich repeat protein 2, neuronal* |
| 27 | *Gng13*  *guanine nucleotide binding protein (G protein), gamma 13* |
| 28 | *Ppap2b*  *phosphatidic acid phosphatase type 2B* |
| 29 | *Not listed* |
| 30 | *Itpr1*  *inositol 1,4,5-trisphosphate receptor 1* |
| 31 | *Nefh*  *neurofilament, heavy polypeptide* |
| 32 | *Atp2a2*  *ATPase, Ca++ transporting, cardiac muscle, slow twitch 2* |
| 33 | *Tm6sf1*  *transmembrane 6 superfamily member 1* |
| 34 | *Nell2*  *NEL-like 2* |
| 35 | *Baiap2*  *brain-specific angiogenesis inhibitor 1-associated protein 2* |
| 36 | *Cacna2d2*  *calcium channel, voltage-dependent, alpha 2/delta subunit 2* |
| 37 | *Ptpn4*  *protein tyrosine phosphatase, non-receptor type 4* |
| 38 | *Cds1*  *CDP-diacylglycerol synthase 1* |
| 39 | *Hapln4*  *hyaluronan and proteoglycan link protein 4* |
| 40 | *Hpca*  *hippocalcin* |
| 41 | *Dgkh*  *diacylglycerol kinase, eta* |
| 42 | *SetD7*  *SET domain containing (lysine methyltransferase)* |
| 43 | *Tex261*  *testis expressed gene 261* |
| 44 | *Prkcg*  *protein kinase C, gamma* |
| 45 | *Plcb4*  *phospholipase C, beta 4* |
| 46 | *Sbk1*  *SH3-binding kinase 1* |
| 47 | *Grik1*  *glutamate receptor, ionotropic, kainate 1* |
| 48 | *Creg1*  *cellular repressor of E1A-stimulated genes 1* |
| 49 | *Lrp8*  *low density lipoprotein receptor-related protein 8, apolipoprotein e receptor* |
| 50 | *LOC270764*  *similar to E2a-Pbx1-associated protein; amyloid-beta precursor protein intracellular domain associated protein 1; cajalin 2* |
| 51 | *Myo10*  *myosin X* |
| 52 | *Tnfrsf21*  *tumor necrosis factor receptor superfamily, member 21* |
| 53 | *Not listed* |
| 54 | *Clmn*  *calmin* |
| 55 | *Gm1399*  *gene model 1399, (NCBI)* |
| 56 | *Bai3*  *brain-specific angiogenesis inhibitor 3* |

Supplementary Table 2.

| **SFARI - High confidence genes** | |
| --- | --- |
| 1 | [*ADNP*](https://gene.sfari.org/database/human-gene/ADNP) |
| 2 | [*ANK2*](https://gene.sfari.org/database/human-gene/ANK2#HG) |
| 3 | [*ARID1B*](https://gene.sfari.org/database/human-gene/ARID1B#HG) |
| 4 | [*ASH1L*](https://gene.sfari.org/database/human-gene/ASH1L#HG) |
| 5 | [*ASXL3*](https://gene.sfari.org/database/human-gene/ASXL3#HG) |
| 6 | [*CHD2*](https://gene.sfari.org/database/human-gene/CHD2#HG) |
| 7 | [*CHD8*](https://gene.sfari.org/database/human-gene/CHD8#HG) |
| 8 | [*CUL3*](https://gene.sfari.org/database/human-gene/CUL3#HG) |
| 9 | [*DSCAM*](https://gene.sfari.org/database/human-gene/DSCAM#HG) |
| 10 | [*DYRK1A*](https://gene.sfari.org/database/human-gene/DYRK1A#HG) |
| 11 | [*GRIN2B*](https://gene.sfari.org/database/human-gene/GRIN2B#HG) |
| 12 | [*KATNAL2*](https://gene.sfari.org/database/human-gene/KATNAL2#HG) |
| 13 | [*KMT2A*](https://gene.sfari.org/database/human-gene/KMT2A) |
| 14 | [*KMT5B*](https://gene.sfari.org/database/human-gene/KMT5B) |
| 15 | [*MYT1L*](https://gene.sfari.org/database/human-gene/MYT1L#HG) |
| 16 | [*NAA15*](https://gene.sfari.org/database/human-gene/NAA15) |
| 17 | [*POGZ*](https://gene.sfari.org/database/human-gene/POGZ#HG) |
| 18 | [*PTEN*](https://gene.sfari.org/database/human-gene/PTEN#HG) |
| 19 | [*RELN*](https://gene.sfari.org/database/human-gene/RELN#HG) |
| 20 | [*SCN2A*](https://gene.sfari.org/database/human-gene/SCN2A#HG) |
| 21 | [*SETD5*](https://gene.sfari.org/database/human-gene/SETD5#GS) |
| 22 | [*SHANK3*](https://gene.sfari.org/database/human-gene/SHANK3#HG) |
| 23 | [*SYNGAP1*](https://gene.sfari.org/database/human-gene/SYNGAP1#HG) |
| 24 | [*TBR1*](https://gene.sfari.org/database/human-gene/TBR1#HG) |
| 25 | [*TRIP12*](https://gene.sfari.org/database/human-gene/TRIP12) |
| **SFARI - Strong candidate genes** | |
| 26 | [*ANKRD11*](https://gene.sfari.org/database/human-gene/ANKRD11) |
| 27 | [*BAZ2B*](https://gene.sfari.org/database/human-gene/BAZ2B) |
| 28 | [*BCKDK*](https://gene.sfari.org/database/human-gene/BCKDK#GS) |
| 29 | [*BCL11A*](https://gene.sfari.org/database/human-gene/BCL11A#HG) |
| 30 | [*CACNA1D*](https://gene.sfari.org/database/human-gene/CACNA1D) |
| 31 | [*CACNA1H*](https://gene.sfari.org/database/human-gene/CACNA1H#HG) |
| 32 | [*CACNA2D3*](https://gene.sfari.org/database/human-gene/CACNA2D3#HG) |
| 33 | [*CIC*](https://gene.sfari.org/database/human-gene/CIC) |
| 34 | *CNOT3* |
| 35 | [*CNTN4*](https://gene.sfari.org/database/human-gene/CNTN4) |
| 36 | [*CNTNAP2*](https://gene.sfari.org/database/human-gene/CNTNAP2#HG) |
| 37 | [*CTNND2*](https://gene.sfari.org/database/human-gene/CTNND2#HG) |
| 38 | *CUX1* |
| 39 | [*DDX3X*](https://gene.sfari.org/database/human-gene/DDX3X) |
| 40 | [*DEAF1*](https://gene.sfari.org/database/human-gene/DEAF1#HG) |
| 41 | [*DIP2C*](https://gene.sfari.org/database/human-gene/DIP2C) |
| 42 | [*ERBIN*](https://gene.sfari.org/database/human-gene/ERBIN) |
| 43 | [*FOXP1*](https://gene.sfari.org/database/human-gene/FOXP1#HG) |
| 44 | [*GABRB3*](https://gene.sfari.org/database/human-gene/GABRB3) |
| 45 | [*GIGYF2*](https://gene.sfari.org/database/human-gene/GIGYF2) |
| 46 | [*GRIA1*](https://gene.sfari.org/database/human-gene/GRIA1) |
| 47 | [*GRIP1*](https://gene.sfari.org/database/human-gene/GRIP1#HG) |
| 48 | [*ILF2*](https://gene.sfari.org/database/human-gene/ILF2) |
| 49 | [*INTS6*](https://gene.sfari.org/database/human-gene/INTS6) |
| 50 | [*IRF2BPL*](https://gene.sfari.org/database/human-gene/IRF2BPL) |
| 51 | [*KAT2B*](https://gene.sfari.org/database/human-gene/KAT2B) |
| 52 | [*KDM5B*](https://gene.sfari.org/database/human-gene/KDM5B#HG) |
| 53 | [*KDM6A*](https://gene.sfari.org/database/human-gene/KDM6A) |
| 54 | [*KMT2C*](https://gene.sfari.org/database/human-gene/KMT2C#HG) |
| 55 | *LEO1* |
| 56 | [*MAGEL2*](https://gene.sfari.org/database/human-gene/MAGEL2#HG) |
| 57 | [*MBOAT7*](https://gene.sfari.org/database/human-gene/MBOAT7) |
| 58 | [*MECP2*](https://gene.sfari.org/database/human-gene/MECP2) |
| 59 | [*MED13*](https://gene.sfari.org/database/human-gene/MED13) |
| 60 | [*MED13L*](https://gene.sfari.org/database/human-gene/MED13L#HG) |
| 61 | [*MET*](https://gene.sfari.org/database/human-gene/MET#HG) |
| 62 | [*MSNP1AS*](https://gene.sfari.org/database/human-gene/MSNP1AS#HG) |
| 63 | [*NCKAP1*](https://gene.sfari.org/database/human-gene/NCKAP1) |
| 64 | [*NLGN3*](https://gene.sfari.org/database/human-gene/NLGN3) |
| 65 | [*NRXN1*](https://gene.sfari.org/database/human-gene/NRXN1#HG) |
| 66 | [*PHF3*](https://gene.sfari.org/database/human-gene/PHF3) |
| 67 | [*PTCHD1*](https://gene.sfari.org/database/human-gene/PTCHD1#HG) |
| 68 | [*RANBP17*](https://gene.sfari.org/database/human-gene/RANBP17) |
| 69 | [*RIMS1*](https://gene.sfari.org/database/human-gene/RIMS1) |
| 70 | [*SCN9A*](https://gene.sfari.org/database/human-gene/SCN9A) |
| 71 | [*SHANK2*](https://gene.sfari.org/database/human-gene/SHANK2#HG) |
| 72 | [*SLC6A1*](https://gene.sfari.org/database/human-gene/SLC6A1) |
| 73 | [*SMARCC2*](https://gene.sfari.org/database/human-gene/SMARCC2) |
| 74 | [*SPAST*](https://gene.sfari.org/database/human-gene/SPAST) |
| 75 | [*SRCAP*](https://gene.sfari.org/database/human-gene/SRCAP) |
| 76 | [*SRSF11*](https://gene.sfari.org/database/human-gene/SRSF11) |
| 77 | [*TAOK2*](https://gene.sfari.org/database/human-gene/TAOK2) |
| 78 | [*TBL1XR1*](https://gene.sfari.org/database/human-gene/TBL1XR1) |
| 79 | [*TCF20*](https://gene.sfari.org/database/human-gene/TCF20) |
| 80 | [*TNRC6B*](https://gene.sfari.org/database/human-gene/TNRC6B) |
| 81 | [*TRIO*](https://gene.sfari.org/database/human-gene/TRIO) |
| 82 | [*UBN2*](https://gene.sfari.org/database/human-gene/UBN2) |
| 83 | [*UPF3B*](https://gene.sfari.org/database/human-gene/UPF3B) |
| 84 | [*USP15*](https://gene.sfari.org/database/human-gene/USP15) |
| 85 | [*USP7*](https://gene.sfari.org/database/human-gene/USP7) |
| 86 | [*WAC*](https://gene.sfari.org/database/human-gene/WAC) |
| 87 | [*WDFY3*](https://gene.sfari.org/database/human-gene/WDFY3) |
