# Supplementary figures and images for: Opposite Expression Patterns of Spry3 and p75NTR in Cerebellar Vermis Suggest a Male-Specific Mechanism of Autism Pathogenesis
Source: Front Psychiatry. 2019 Jun 18;10:416. doi: 10.3389/fpsyt.2019.00416 (PMC6591651; doi:10.3389/fpsyt.2019.00416)

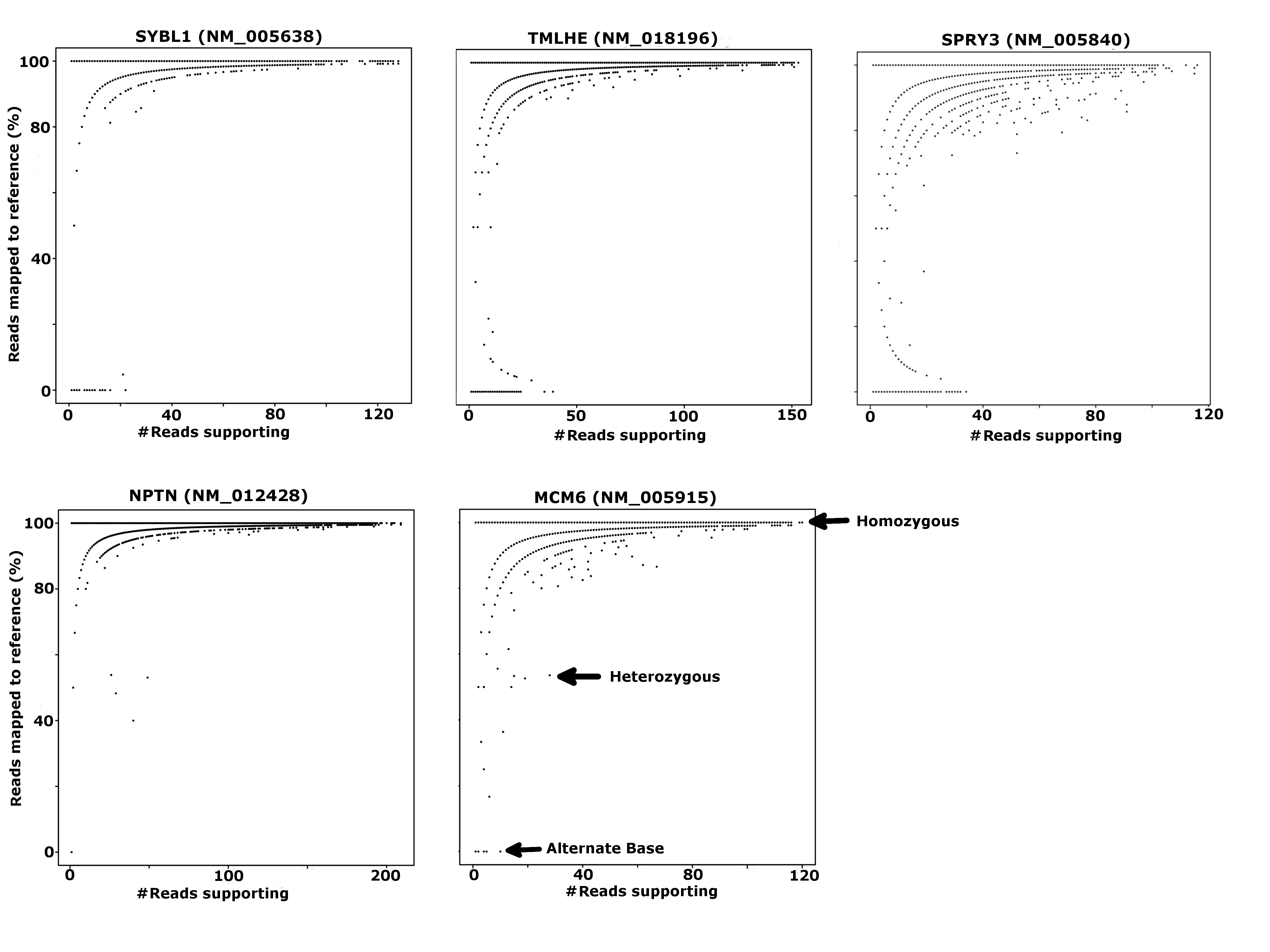

Supplement: Supplementary Figure 1 — Comparison of SPRY3 expression heterozygosity map with maps for TMLHE (X-linked), SYBL1 (PAR2-linked), and NPTN, MCM6 (autosomal) genes. [file Image_1.tif]
